# The Institutional Library

**Published:** 1913-08-02

**Authors:** 


### THE INSTITUTIONAL LIBRARY.

A Text-book of Midwifery. By R. W. Johnstone, M.D., F.R.C.S. (London: A. and C. Black. 1913. Pp. 485. Price 10s. 6d. net.)

This new text-book of midwifery follows mainly along accepted and familiar lines, and should become popular with medical students. It is, we should say, not quite large enough and detailed enough in the practical parts for practitioners who want to master the less elementary principles of the obstetrical art and science. The sections on embryology, anatomy of the pelvis, mechanisms, and conduct of labour are all very sound and unexceptionable, as, indeed, they would be in any book which emanates from a teacher of Dr. Johnstone's experience and reputation. In dealing with the toxæmias of pregnancy a good deal of reserve is maintained; but this is quite a necessary piece of prudence in the present limited state of knowledge. We note that the author is one of those who favour rapid delivery in eclampsia, and, for our own part, we quite agree, though a good many respected authorities hold otherwise. Vaginal hysterotomy is recommended in preference to other forms of *accouchement forcé* for this condition. We are somewhat surprised that no mention is made of Cæsarean section in the treatment of central placenta prævia; and we decline to believe that, taking in this form as well as the lateral and marginal varieties, placenta prævia is anything like so rare as one in a thousand cases. It is true that many of the marginal cases cause symptoms so slight as to be almost negligible; but, for all that, they are comparatively common, though often unrecognised. Taking the book all round, it is a creditable effort, and the publishers have done their share in making it attractive.

Abel's Laboratory Handbook of Bacteriology. Second English Edition. Translated by M. H. Gordon, M.A., M.D. With additions by Dr. Houston and Dr. Horder. (London: Henry Frowde and Hodder and Stoughton. 1913. Price 5s. net.)

The small compass of this book is deceptive; its modest size bears no proportion to the relatively enormous amount of practical detail contained in it. Like its German parent, which has already run into fifteen editions, this English edition is concise, not to say concentrated, at the expense of clearness. As a laboratory manual, and not a text-book for systematic reading, this volume is one of the best of its kind. The methods described are those in actual use in any large laboratory, but some regard is paid to the needs of the student and the beginner in practical bacteriology. The sections by Dr. Houston on the examination of water, milk, and sewage, and the translator's description of the bacteriological examination of dust and air, will appeal to workers for the diploma in public health.

Studies in Smallpox and Vaccination. By Wm. Hanna, M.A., M.D., D.P.H. (Bristol: John Wright and Sons, Ltd. 1913.)

This monograph possesses more practical interest than is usually associated with this class of medical literature. Dr. Hanna has dealt with a problem which may at any time become one of pressing importance, and has presented his facts and deductions in a laudably clear and direct fashion. Even without the letterpress, the excellent and very numerous photographs with the lucid charts and tables are sufficient to impress the reader with the undoubted value of vaccination, not only as a protection against smallpox, but also as a potent means of modifying the attack when this operation is performed during the incubation period. This monograph is indeed an object-lesson for the doubters and the "anti's," and it would be extremely interesting to watch any attempt by the anti-vaccination societies to get round Dr. Hanna's observations. Very complete analyses are given bearing on the relation between scar area and severity of the disease, as well as statistics of the modifying influence of vaccination in infancy, in adults, and concurrently with variola. Dr. Hanna finds that among contacts the cases which show signs of the vaccination "taking" are just those which require to be watched and isolated, rather than those in which the vaccination is not proving successful—an interesting statement and one of great practical importance for quarantine officials. The opposite view is one which has up to the present been the more frequently expressed.

### VOLUNTARY AID.

Manual for Women's Voluntary Aid Detachments. By Lieut.-Col. P. C. Gabbett, M.R.C.S. (Bristol: J. Wright and Sons. Second Edition. 1913. Pp. 115. Price 1s. net.)

First Aid to the Injured and Sick. By F. J. Warwick, M.B., and A. C. Tunstall, M.D. (Bristol: J. Wright and Sons. Eighth Edition. 1913. Pp. 246. Price 1s. net; leather, 2s. 6d.)

We are in full agreement with Colonel Gabbett's sensible remarks concerning the proper functions of the Women's Voluntary Aid Detachments of the Territorial Scheme and with his advice against their attempting to learn those transport and similar duties which are properly the functions of the men's detachments. In this splendid little manual he presupposes that his pupils have already passed courses both of first aid and of home nursing; and proceeds to instruct them in rather more advanced military nursing on that foundation. As he rightly remarks, only actual experience of operations and similar serious cases can really qualify the amateur nurse for efficient service in a V.A.D.; and he accordingly urges all who are properly keen on their work to get some practical training in a hospital. This counsel of perfection we very heartily second; but we are bound to admit that there are considerable difficulties to be surmounted, unless the amateur nurse is prepared to turn herself into a professional one and enter for a regular course of training. At all events, we willingly concede that Colonel Gabbett's book is bound to help materially in the training of all V.A.D. members who master its contents; and we are not in the least surprised that it has already reached a second edition.

Drs. Warwick and Tunstall's well-known First Aid Manual continues its triumphant career of fresh editions, and seems likely to do so for a long time. Successive revisions have resulted in a text-book than which we know no better. It is, of course, somewhat more advanced than the much smaller St. John's Ambulance official book compiled by Mr. Cantlie; but it can quite easily be used as a class-book for the most elementary lectures by a judicious selection of its contents. We cannot say more than that each new edition increases our respect for this comprehensive and common-sense manual.

